# Influence of wound fluid on chemotherapy sensitivity in primary breast cancer cells

**DOI:** 10.18632/oncotarget.11345

**Published:** 2016-08-17

**Authors:** Yingchao Zhang, Dong Yan, Hao Zhang, Xunyan Ou, Zuowei Zhao, Dan Wang, Caigang Liu

**Affiliations:** ^1^ Department of Breast Surgery, The Second Hospital of JiLin University, Changchun, 130021, China; ^2^ Department of Pediatrics, The Second Hospital of Dalian Medical University, Dalian, 114006, China; ^3^ Breast Disease and Reconstruction Center, Breast Cancer Key Laboratory of Dalian, The Second Hospital of Dalian Medical University, Dalian, 114006, China

**Keywords:** breast cancer, wound fluid, chemotherapy, drug resistance

## Abstract

**Objective:**

To investigate the effect of WF on chemotherapy sensitivities of primary breast cancer cells from breast cancer patients by using CD-DST.

**Results:**

In general, the WF-treated cells showed remarkable increase in survival rates as compared to the control cells cultured without WF among different anticancer drug subgroups. This trend was generally observed in all the tumor cells from the premenopausal, postmenopausal, T2, N0, N1, luminal B, and TN patients.

**Methods:**

The sensitivities of WF-treated primary breast cancer cells, from 21 patients who underwent a radical resection for breast cancer from September 2014 to July 2015, to anticancer drugs: EPI, CDDP, DOC, VNR, 5-FU+LV, and PAC, were obtained using CD-DST. The survival rates of the breast cancer cells were recorded and used to gauge the chemotherapeutic effect.

**Conclusions:**

Surgery-induced WF promotes the drug resistance of primary breast cancer cells to chemotherapy, suggesting that surgery may have adverse effects on breast cancer patients. More studies are needed to investigate the key factors in WF that enhance the susceptibility to chemotherapy drugs.

## INTRODUCTION

Breast cancer is the most common cause of death in female malignant tumor disease [1]. Chemotherapy is the most important systemic adjuvant therapy for breast cancer that still has an irreplaceable role [2]. However, the efficacy of chemotherapy is often severely affected by intrinsic and acquired drug resistance [3]. The tumor cells of patients with different clinical and pathological features have different susceptibility to chemotherapy. Further, the tumor cells of patients with different features also have different susceptibility to various chemotherapy drugs. In the process of chemotherapy, it is difficult to implement clinically a personalized and precise treatment plan for each patient. Predicting the chemosensitivity of patients with malignant disease to anticancer drugs may help to avoid the use of ineffective anticancer drugs [4].

*In vitro* anticancer drug sensitivity tests using clinical specimens are representative modalities for obtaining useful data for designing individualized chemotherapy [5]. The collagen gel droplet embedded culture drug test (CD-DST) is an *in vitro* anticancer drug sensitivity test that has been applied for chemotherapy of breast and non-small cell lung cancer patients [6, 7].

In our previous study, we demonstrated that post-surgery WF promotes the proliferation and migration of breast cancer cells and that the proliferative effect is concentration-dependent to a certain extent, which supports the previous findings that surgery may have adverse effects on breast cancer patients [8]. We conducted this study to obtain a preliminary conclusion for patients who received chemotherapy sensitivity testing, as well as to observe the susceptibility of the primary tumor cells from patients with different features to various chemotherapy drugs. In addition, we co-cultured the tumor cells of some patients with their respective postoperative wound fluid (WF) to observe the effects of WF on drug susceptibility. Our results are consistent with previous studies and provide interesting insights into how chemotherapy for breast cancer patients can be implemented in a personalized and precise manner.

## RESULTS

### Effects of WF on drug resistance of primary breast cancer cells in the general patient population

**Figure 1:**
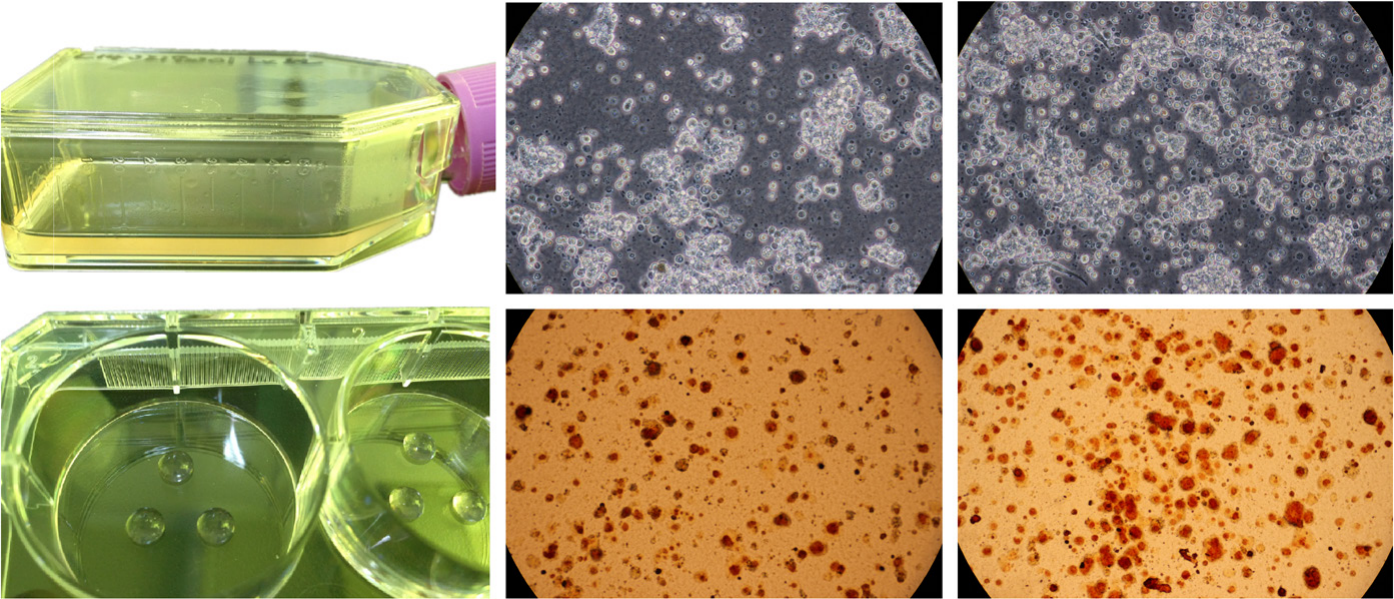
The primary breast cancer specimen was cut into small pieces and was digested in a cell dissociation enzyme solution EZ Then the cells were pre-cultured in CG flasks. Cancer and fibroblast cells showed specific growth morphologies in the collagen gel droplets. The anticancer agents were added at physiological concentration, and the cells were stained with neutral red and fixed with neutral formalin buffer.

**Figure 2:**
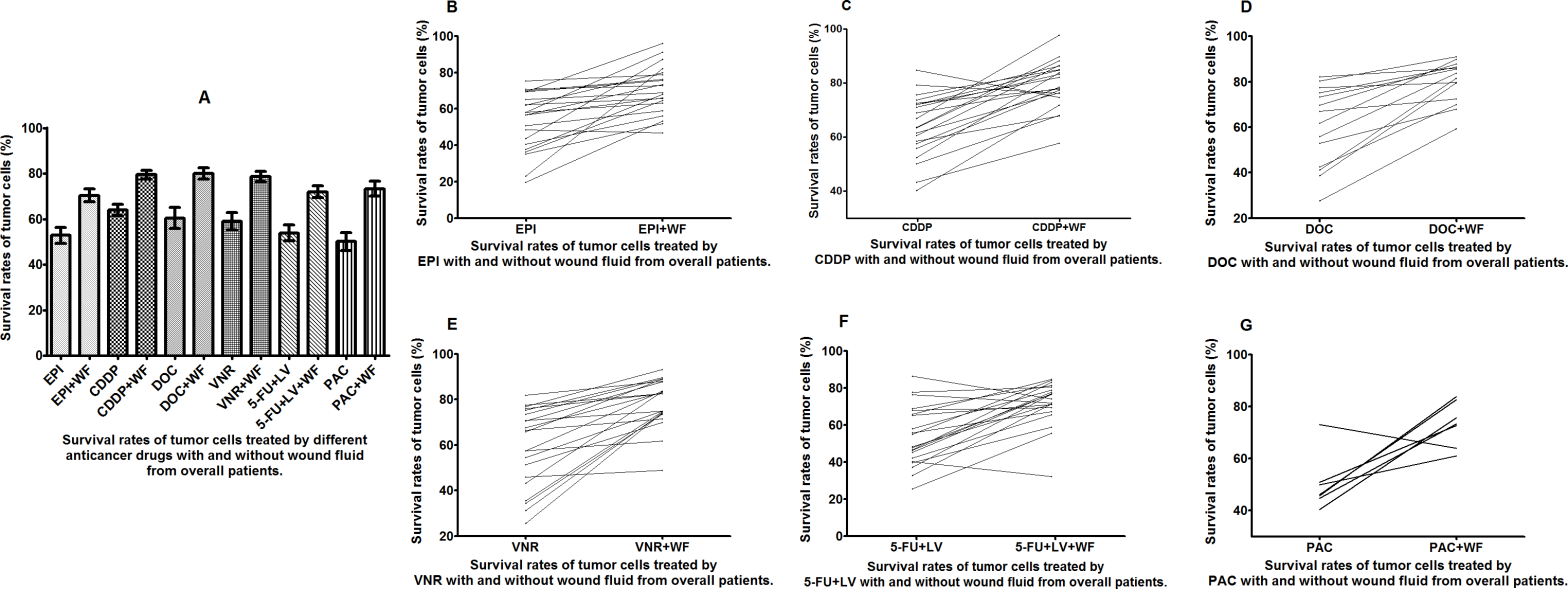
Survival rates of untreated and WF-treated tumor cells from overall patients after culture with different anticancer drugs (**A**) Survival rates of untreated and WF-treated tumor cells after culture with EPI (**B**), CDDP (**C**), DOC (**D**), VNR (**E**), 5-FU+LV (**F**), and PAC (**G**).

The primary breast cancer cells from patients were cultured with or without WF, and after treating with different anticancer drugs, survival rates were assessed *in vitro* by performing CD-DST (Figure 1). Generally, the WF-treated cells showed remarkable increase in survival rates as compared to the control cells cultured without WF among the different anticancer drug subgroups (Figure 2).

### Effects of WF on drug resistance of breast cancer cells in patients with different menopausal status

**Figure 3:**
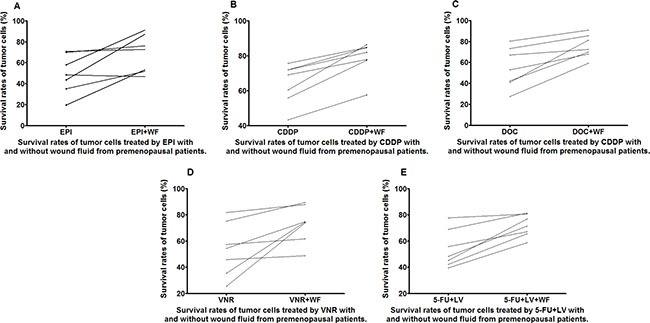
Survival rates of untreated and WF-treated tumor cells from premenopausal patients after culture with the anticancer drugs EPI (A), CDDP (B), DOC (C), VNR (D), and 5-FU+LV (E)

**Figure 4:**
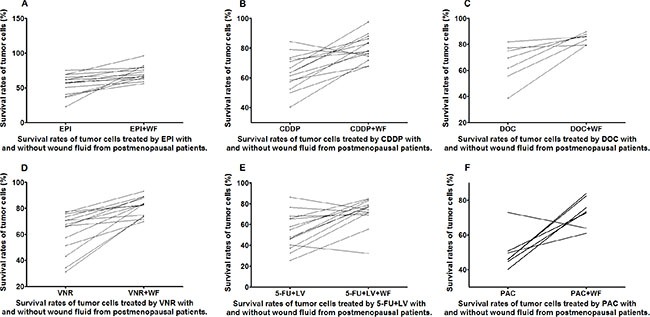
Survival rates of untreated and WF-treated tumor cells from postmenopausal patients after culture with different anticancer drugs EPI (A), CDDP (B), DOC (C), VNR (D), 5-FU+LV (E), and PAC (F)

The WF-treated cells showed remarkable increase in survival rates as compared to the control cells cultured without WF among the different anticancer drug subgroups in both pre- and postmenopausal patients (Figures 3 and 4). It is worth noting that the survival rates of WF-treated breast cancer cells from premenopausal patients were significantly higher than those of control group for all the anticancer drugs.

### Effects of WF on drug resistance of breast cancer cells in patients with different tumor stages

**Figure 5:**
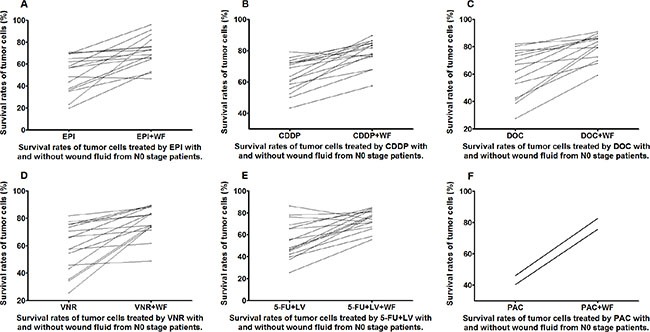
Survival rates of untreated and WF-treated tumor cells from N0 patients after culture with different anticancer drugs EPI (A), CDDP (B), DOC (C), VNR (D), 5-FU+LV (E), and PAC (F)

**Figure 6:**
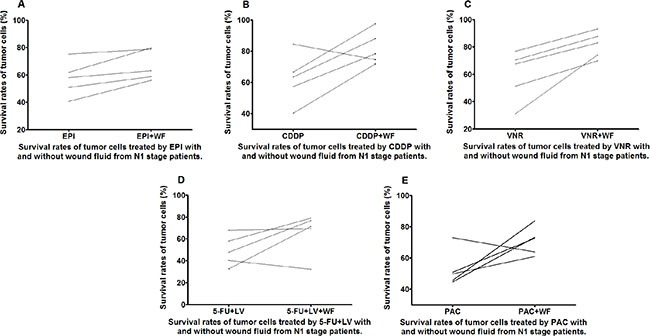
Survival rates of untreated and WF-treated tumor cells from N1 patients after culture with different anticancer drugs EPI (A), CDDP (B), VNR (C), 5-FU+LV (D), and PAC (E)

The WF-treated cells showed increase in survival rates as compared to the control cells cultured without WF among the different anticancer drug subgroups in both the N0 and N1 stage patients (Figures 5 and 6).

### Effects of WF on drug resistance of breast cancer cells in patients with different molecular subtypes

**Figure 7:**
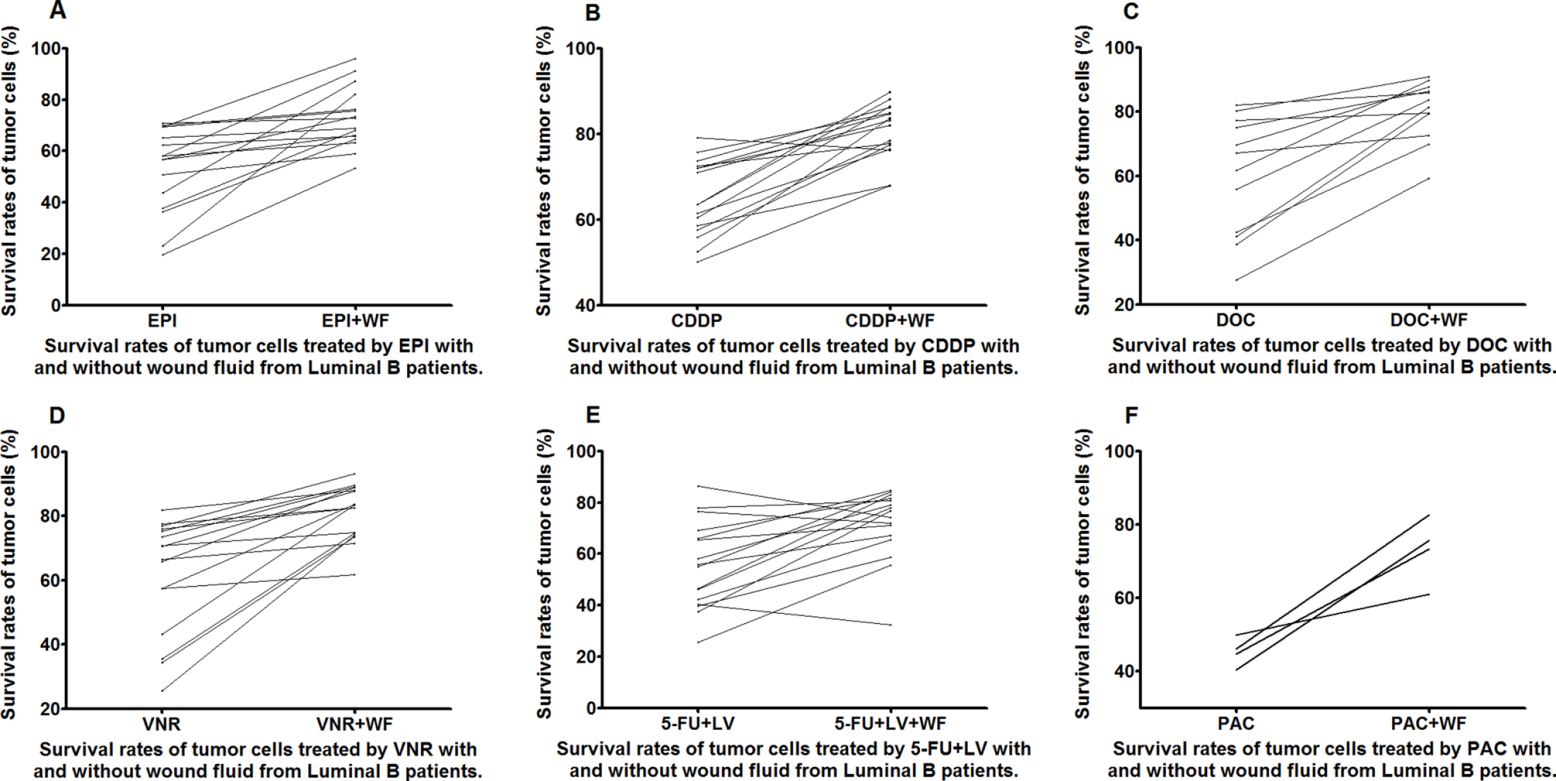
Survival rates of untreated and WF-treated tumor cells from luminal B patients after culture with different anticancer drugs EPI (A), CDDP (B), DOC (C), VNR (D), 5-FU+LV (E), and PAC (F)

**Figure 8:**
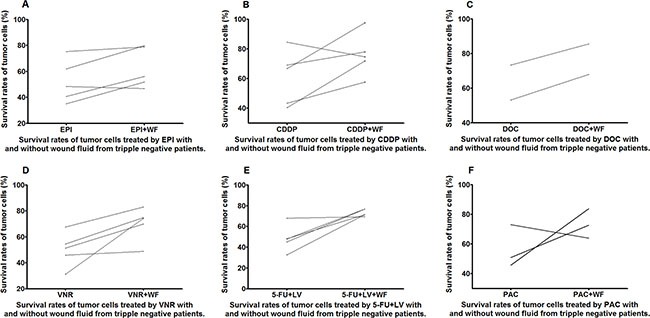
Survival rates of untreated and WF-treated tumor cells from TN patients after culture with different anticancer drugs EPI (A), CDDP (B), DOC (C), VNR (D), 5-FU+LV (E), and PAC (F)

Almost all the WF-treated cells showed increase in survival rates as compared to the control cells cultured without WF among the different anticancer drug subgroups in both luminal B and tripple negative (TN) patients (Figures 7 and 8).

## DISCUSSION

In this study, we found that WF promotes drug resistance of primary breast cancer cells to chemotherapy, which is similar to our previous results that WF increased proliferation and migration of breast cancer cells. The significance of co-culture with WF suggested that in line with the doctrine of tumor microenvironment, cytokines in the WF provide a better microenvironment for the tumor cells, thereby enhancing proliferation and drug resistance of the tumor cells.

The survival rates of the primary tumor cells changed between the untreated control and WF-treated groups. Overall, we can see that after the addition of WF, the tumor cells exhibited enhanced resistance to all the chemotherapy drugs (Figure 2A). Looking in particular at the line graphs showing the survival rates of tumor cells in each anticancer drug subgroup, in which the samples on the left are the untreated cells and on the right the WF-treated cells, we can observe an upward trend from left to right that represents an increase in the drug resistance of the tumor cells (Figure 2B–2G).

We analyzed the susceptibility of tumor cells from pre- and postmenopausal women, patients with T2–N1 stage breast cancer, and patients with luminal B and tripple negative breast cancer to different chemotherapy drugs.

The drug resistance of the primary tumor cells derived from premenopausal patients increased significantly after the addition of WF, which is apparently very detrimental to a good prognosis. However, it should also be noted that the tumor cells from these patients were susceptible to the chemotherapy drugs before the addition of WF. This phenomenon is consistent with our general clinical understanding that younger premenopausal patients are the population that is at high risk [11].

The change in the survival rates of the primary tumor cells from postmenopausal patients was relatively insignificant compared to the premenopausal patients, as evident from the slopes of the lines in the graphs. The tumor cells from postmenopausal patients were also correspondingly less susceptible to the chemotherapy drugs [12]. The drug susceptibility of tumor cells from N0 and N1 patients is similar to those from postmenopausal patients. In addition, the tumor cells from N0 patients are more susceptible than those from N1 patients, which may lead to a difference in prognostic outcome between the N0 and N1 patients [13].

The tumor cells from hormone receptor-positive patients tend to be less susceptible to chemotherapy, especially those from patients with luminal A breast cancer. However, the tumor cells from patients with luminal B breast cancer showed a slightly better susceptibility [14] and chemotherapy is still the most commonly used form of systemic adjuvant therapy for patients with triple negative breast cancer [15]. The tumor cells from patients with luminal B breast cancer are susceptible to various chemotherapy drugs. However, the tumor cells with good drug susceptibility often have higher drug resistance upon the addition of WF, which is similar to the situation observed in the premenopausal patients, and the tumor cells from patients with triple negative breast cancer are less susceptible to chemotherapy drugs even before the addition of WF, suggesting higher drug resistance.

Therefore, we need to increase the sample size, conduct follow-ups, and carry out a more detailed stratification research on the patients to screen for patients that are more like to respond better to chemotherapy, in order to implement a more precise treatment. Likewise, we should also study the key factors in WF that enhance the susceptibility to chemotherapy drugs.

In conclusion, the tumor cells from premenopausal patients with luminal B breast cancer have a better overall susceptibility to chemotherapy. Some postmenopausal, N0, and N1 patients are still relatively susceptible to certain chemotherapy drugs, but the WF had an adverse effect on their drug resistance.

## MATERIALS AND METHODS

### Patients and WF collection

A total of 21 patients, who had undergone mastectomy with axillary dissection for breast cancer, were enrolled at the Second Hospital of Dalian Medical University, China between September 2014 and July 2015 Table 1. All patients had no underlying diseases except breast neoplasm. Written informed consent was obtained from individual patients, and the experimental protocol was approved by the Ethics Committee of Dalian Medical University.

**Table 1:** Clinicopathological features of patients enrolled in CD-DST (*n* = 21)

Variables	*N*
**Menstrual condition**	
Pre-menopause	7
post-menopause	14
**NAC**	
Yes	12
No	9
**Tumor size**	
T2	21
**N stage**	
N0	16
N1	5
**ER status**	
negative	5
positive	16
**PR status**	
negative	14
positive	7
**Her-2 status**	
negative	12
positive	9
**Ki-67 status**	
> 14%	21
**Molecular subtype**	
Luminal B	16
Tripple negative	5

A total of 50 ml wound drainage fluids were collected in a sterile container without any additives from individual patients by placing the perforated end of the surgical drain in the chest wall and/or axilla wounds on day 3 post surgery. After being centrifuged at 1,600 RCF for 10 minutes, the concentrations of total proteins in the supernatants of WF samples were determined by the bicinchoninic acid (BCA) assay using the Pierce *BCA Protein* Assay Kit, according to the manufacturers' instruction (Fisher Scientific, Canoga Park, USA). The mean concentrations of total proteins in the WF samples were 2.61 ± 1.2 mg/ml. Individual WF supernatant samples were aliquoted and stored at −80°C until use in CD-DST.

### CD-DST data acquisition

CD-DST was performed as previously described by Kobayashi et al. [9, 10]. In brief, each fresh specimen obtained by surgery was finely minced using a scalpel and digested in a cell dispersion enzyme solution (EZ, Nitta Gelatin Inc., Osaka, Japan) for 2 h. The dispersed cancer cells were washed twice, collected by centrifugation at 250 ×*g* for 3 min, filtered through an 80 um nylon mesh, and then incubated in a collagen gel coated flask (CG-flask, Nitta Gelatin Inc.) in a CO_2_ incubator at 37°C for 24 h. Only the viable cells adhering to the collagen gel were collected and suspended in the reconstructed type I collagen solution (Cellmatrix Type CD, Nitta Gelatin Inc.) with the final density being 1 × 10^5^ cells/ml. Three drops of the collagen-cell mixture (30 μl/drop) were placed in each well of a 6-well multiplate and in a 60-mm dish, and allowed to gel at 37°C in a CO_2_ incubator for 1 h. The final concentration was about 3 × 10^3^ cells/collagen gel droplet. The culture medium with or without 1% WF was overlaid on each well, and the plate was incubated in a CO_2_ incubator at 37°C overnight. The anticancer drug was added, and incubated for 1 h (gemcitabine) or 24 h (other drugs). After the removal of the medium containing anticancer drugs, each well was rinsed twice, overlaid with serum-free culture medium (PCN-1, Nitta Gelatin Inc.), and incubated for 7 days. On the fourth day of the incubation, the medium was changed once. At the end of incubation, neutral red was added to each well at a final concentration of 50 μg/ml, and the colonies in the collagen gel droplets were stained for 3 h. Collagen droplets in the 60 mm dish were stained just before exposure (day 1). Thereafter, each collagen droplet was fixed with 10% neutral formalin buffer, washed in water, air dried, and quantified by image analysis. A tumor cell survival rate less than 50% was regarded as *in vitro* sensitive.

### Anticancer drugs

The anticancer drugs tested in the CD-DST were 0.78 μg/ml epirubicin (EPI), 0.1 μg/ml cisplatin (CDDP), 0.2 μg/ml docetaxel (DOC), 0.1 μg/ml vinorelbine (VNR), 0.05 μg/ml fluorouracil + 5.0 μg/ml leucovorin (5-FU+LV), and 1.0 μg/ml paclitaxel (PAC). The culture time was 24 h for each drug.

### Statistical analysis

Relationships between the two groups (with or without WF) and other parameters were studied using the Pearson's Chi-square test or Fisher's exact test. Paired *T*-test was used to assess differences between the two groups.

Two-sided *P* values were calculated for all tests and are reported here. *P* values less than 0.05 were considered to indicate statistical significance.

Analyses were performed with the use of SPSS software (version 19.0; SPSS, Inc., Chicago, IL, USA). The computer program PRISM (version 5; GraphPad Inc., San Diego, CA, USA) was used to create graphs and process images.

## Abbreviations

WF: wound fluid
CD-DST: collagen gel droplet embedded culture drug test
EPI: epirubicin
CDDP: cisplatin
DOC: docetaxel
VNR: vinorelbine
5-FU+LV: fluorouracil + leucovorin
PAC: paclitaxel.
